# Priority Effects of Time of Arrival of Plant Functional Groups Override Sowing Interval or Density Effects: A Grassland Experiment

**DOI:** 10.1371/journal.pone.0086906

**Published:** 2014-01-31

**Authors:** Philipp von Gillhaussen, Uwe Rascher, Nicolai D. Jablonowski, Christine Plückers, Carl Beierkuhnlein, Vicky M. Temperton

**Affiliations:** 1 Institute of Bio- and Geosciences: Plant Sciences (IBG-2), Forschungszentrum Jülich GmbH, Jülich, Germany; 2 Department of Biogeography, University of Bayreuth, Bayreuth, Germany; Centro de Investigación y de Estudios Avanzados, Mexico

## Abstract

Priority effects occur when species that arrive first in a habitat significantly affect the establishment, growth, or reproduction of species arriving later and thus affect functioning of communities. However, we know little about how the timing of arrival of functionally different species may alter structure and function during assembly. Even less is known about how plant density might interact with initial assembly. In a greenhouse experiment legumes, grasses or forbs were sown a number of weeks before the other two plant functional types were sown (PFT) in combination with a sowing density treatment. Legumes, grasses or non-legume forbs were sown first at three different density levels followed by sowing of the remaining PFTs after three or six-weeks. We found that the order of arrival of different plant functional types had a much stronger influence on aboveground productivity than sowing density or interval between the sowing events. The sowing of legumes before the other PFTs produced the highest aboveground biomass. The larger sowing interval led to higher asymmetric competition, with highest dominance of the PFT sown first. It seems that legumes were better able to get a head-start and be productive before the later groups arrived, but that their traits allowed for better subsequent establishment of non-legume PFTs. Our study indicates that the manipulation of the order of arrival can create priority effects which favour functional groups of plants differently and thus induce different assembly routes and affect community composition and functioning.

## Introduction

Research into the assembly of ecological communities has shown that the extant composition of communities is strongly influenced by historical factors [1]–[3]. Priority effects occur in communities, when one (or more) species already is present in a habitat and thereby affects the success of later species [4], [5], and this effect can be either negative, positive or neutral [6]. The success of other species can relate to their establishment, growth or reproduction [7]. Priority effects are thus important e.g. to understand when applying ecological theory and knowledge to help restore degraded habitats where certain species are introduced to a site via restoration (Grman and Suding 2010). Species arriving prior to other species are generally considered to either affect newcomers via size-asymmetric competition [8] or so-called legacies in the soil created by effects of plant-soil feedback on the soil [9]. Another possible mechanism of priority effects is nitrogen (N) facilitation (including N transfer and N sparing) between N_2_-fixing species arriving early during assembly and other functionally different species arriving at a later time-point (see Körner et al. [10] for first indication of this). No matter the mechanism, the outcome of priority effects seems to be that competitive and or facilitative interactions for newcomers are altered.

Priority effects can lead to lasting differences in species or functional composition, and hence can potentially drive ecosystem properties and functioning, and may sometimes even have a stronger influence than the effects of abiotic conditions on community composition [1], [11]. In aquatic model-ecosystems also, there is evidence that properties, such as biomass production or community size, seem to be more dependent on initial arrival order and frequency than on other factors such as initial species richness [4], [12]. Recent research has found a mediating role of soil resource availability in relation to the importance of priority effects, however, at least in a pot experiment [13].

Recent research has focused on two different kinds of priority effects in plant assembly, the one showing long-term effects on vegetation caused by adding species mixtures at the same time [14], [15] or altering the sequence of arrival of different species or groups of species [10], [11], [13]. Although the simultaneous introduction of species is of high relevance to restoration projects where mixtures of plants are often used, the potential mechanisms of order of arrival of in particular different functional groups has not been much explored yet. N_2_-fixing legumes are known to be ecosystem engineers, in particular introducing extra N_2_ into soils and hence driving N cycling and community productivity [16]. We now know from many biodiversity experiments that niche complementarity between species varying in traits can lead to better overall resource-use at community level, and that particular combinations of functional groups (particularly N_2_-fixers combined with grasses) as well as species richness can drive positive diversity effects [17]–[20]. It may be that this complementarity between different functional groups is a driver of assembly over time, and hence composition and functioning of communities.

Körner et al. (2008) varied the arrival order of three different plant functional types (from hereon called PFTs) each containing two out of six plant species in microcosms, with either legumes, non-legume forbs, or grasses sown first and the other two groups sown three weeks later. They found strong priority effects of sowing legumes first on both aboveground and belowground community productivity, even after two growing seasons. In their study the set of species in each microcosm was comparably small in relation to the biodiversity of common grasslands in central Europe. To be able to set the outcome of such a study into a more applied context (e.g. restoration or creation of semi-natural grasslands) it is essential to look on the species which occur naturally in such environments. In particular to enhance restoration of species-rich grasslands, the role of legumes as possible ecosystem engineers on nutrient-poor soils needs further research.

As the number of species in a system increases so does the number of possible interactions, either positive or negative thus affecting assembly [21]–[23]. We know from many biodiversity experiments that niche complementarity between species varying in traits can lead to better overall resource-use at community level, and that particular combinations of functional groups (particularly N_2_-fixers combined with grasses) as well as species- and functional group richness can drive positive diversity effects [17]–[20].

Species that arrive first at a site have a competitive advantage over those that arrive later, and the longer the time interval between establishment episodes the more asymmetric competition may become [8]. The relative benefit one PFT can get through this competitive advantage of arriving first, however, might become a benefit for the whole community when these species have special traits such as legumes due to their ability to increase N availability either via N sparing or via N transfer. Therefore especially in harsh environments (e.g. low initial nutrient content or high environmental stress) legumes may have a positive effect not only on productivity but also on other species survival and establishment and thus positively influencing assembly [24]. Positive effects found on productivity by sowing legumes before other functional groups [10] were related to a three-week sowing interval. To what extent the sowing interval affects assembly outcomes now needs further study, since the ontological state (life stage) of a plant population may influence the species interactions and hence priority effects.

Community assembly in general and priority effects are in all likelihood modulated by both density of individuals in communities as well as environmental resource availability [8]. The law of constant yield predicts that even-aged populations grown in different densities show the same overall productivity after a certain period of time [25]. Where initial biomass is higher with increasing density this relationship wears off with time leading to the same productivity of standing biomass independent of the population density (with higher individual numbers in high densities but lower standing biomass per individual). Competition for nutrients is considered the key mechanism behind the constant yield law, but size-density relationships may change in different environments [26]. Under more extreme environmental conditions, for example, facilitation may drive communities as much as competition does. The size of an individual does not necessarily decrease with increasing density. Indeed, if facilitation and competition take place simultaneously, the size of individuals may even increase with density. In addition, sowing at high densities is often associated with higher cover values and relative abundance of sown species [27], [28] correlating with greater productivity. In this sense increasing sowing density could potentially have a positive effect on productivity. However size-density-yield relationships especially in mixed stands have rarely been investigated.

This study investigated the effect of order of arrival (priority effect) of functionally different species groups (PFTs) on the productivity as well as species and functional composition of species-rich grassland communities grown in pots under greenhouse conditions. The experiment was multi-factorial regarding order of arrival, density and sowing interval as factors tested for their effects on community productivity and composition. The following hypotheses were tested:

1. The longer the sowing interval between the PFT sown first and the subsequently sown PFTs the lower the aboveground productivity of the system will be. This is due to stronger asymmetric competition between PFTs when early arrivers get a head-start and very little complementarity between PFTs can occur.
2. Sowing different seed densities will result in higher individual numbers at higher sowing densities but overall aboveground productivity will remain the same across all levels of the density treatment due to the law of constant yield.

## Materials and Methods

### Experimental Setup and Initial Conditions

A pot experiment was set up in the greenhouse of the Institute of Bio- and Geosciences (IBG-2), Germany in April 2011 sowing seeds typical of mesic and dry grassland habitats in the region. The experiment lasted from May until August (a total of 18 weeks from first sowing to harvest). A total of 28 typical central European grassland species were selected belonging to the three plant functional types forbs, grasses and legumes (PFT: 14 forb-, 7 grass-, 7 legume species; for species list see Supporting Information, Table. S1: Plant species per functional group with respective seed mass per pot). We chose this relative contribution of the three PFTs based on relative abundances in natural or semi-natural communities in such grasslands in Germany, (Matthias Solle, personal communication) known to have different effects on nutrient cycling and productivity from biodiversity experiments [18], [29]. Species selection was based on broad phytosociological units of the given grassland communities in dry to mesic conditions [30] and Ellenberg's indicator values [31].

### Experimental Design

Pots with a volume of 5 litre and an upper diameter of 20 cm and a diameter of 15 cm at the bottom were filled with a 1∶2 mixture of sand (grain size 0.7–1.4 mm) and low nutrient potting soil (Einheitserde- und Humuswerke GmbH & Co. KG; “Typ P”) as a substrate (for initial nutrient status of the soil see Supporting Information, Table. S2: Results of soil analysis at the beginning of the experiment). By using a substrate with low initial nutrient status we wanted to foster effects of positive and negative plant-plant interaction to be reflected in productivity and species composition. Sand was added to increase water permeability.

**Figure 1:**
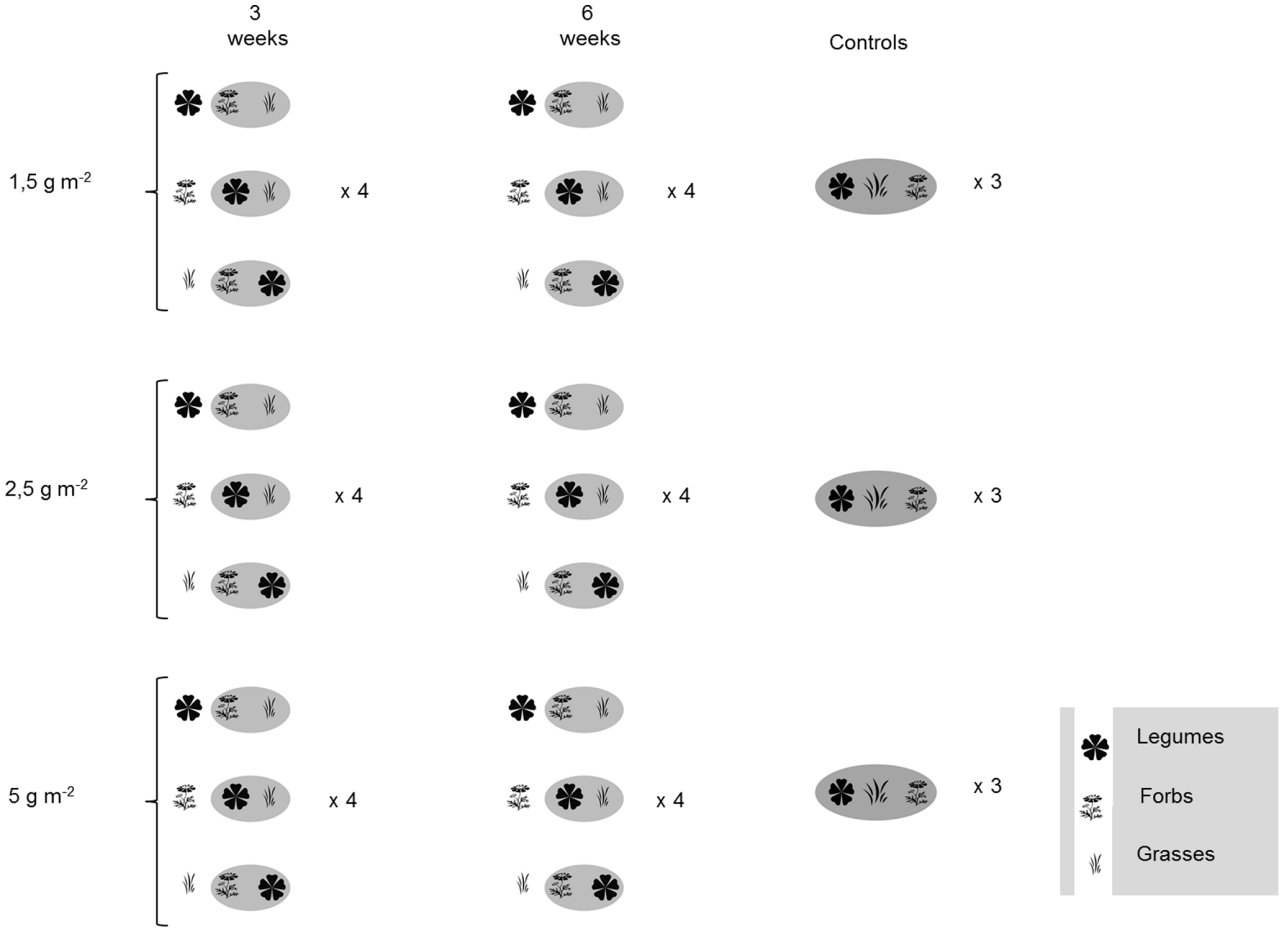
Experimental setup showing the three different treatments of the experiment: the priority effect treatment of arrival order, the different density treatment and the sowing interval treatment. Priority effects of early sowing of one PFT are shown with a plant functional group symbol without a circle, and later sowing of the remaining two PFTs are shown in grey circles. For the priority effect treatment legumes, forbs or grasses were sown a number of weeks before the other two groups. Density levels were 1.5, 2.5 and 5^−2^, and sowing intervals were three- and six-weeks between first PFT sown and remaining PFTs. Controls involved all PFTs being sown together at the same time. Number of replicates is shown in bold next to each treatment.

Plant species density was standardised on behalf of their seed mass (giving a sowing density) and records of germination capacity. Seeds were obtained from Rieger-Hofmann GmbH and mixed manually to form a density treatment with three different levels (1.5; 2.5 & 5 g m^−2^). Before sowing, densities were calculated on the basis of the thousand-seed weight (*TSW*) of each of the species (for species list see Supporting Information, Table. S1: Plant species per functional group with respective seed mass per pot) and an empirical value derived from germination tests (*A*) standing for number of individuals of species “x” m^−2^ (Matthias Stolle, Rieger-Hofmann GmbH, personal communication) for pot surface area (*B*) and a factor (*Y*) to meet the desired plant density level, as follows:
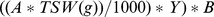



Assembly order was influenced through a variation in order of arrival (sowing time) of three different PFTs. Species groups referred to as PFTs were non-legume forbs (hereafter referred to as forbs), nitrogen-fixing Fabaceae (legumes) and grasses. Four priority effect treatments (PE) were set up: forbs sown first (F-first), grasses sown first (G-first), legumes sown first (L-first) and a control treatment with all PFTs sown together at the same time. The priority effect treatment was created by sowing one PFT first on one sowing date (13-April-2011) and the other two remaining PFTs respectively three-(04-May-2011) or six weeks (25-May-2011) later providing a sowing interval treatment of either three- or six weeks. Each priority effect- and sowing interval treatment was additionally sown at three different density levels giving four replicates per PE-, density- and sowing interval-treatment (Fig. 1).

Pots were watered continuously by an automated irrigation system (Gardena) using rain water. Water was allowed to drain from the pots through holes in the bottom. Temperatures in the greenhouse varied from 17°C at night to 25°C in the day during the experimental period. Sowing occurred in all 81 pots one week after the filling of to allow the substrate to rest. Three soil samples were taken at time zero to evaluate the nutrient status at the beginning of the experiment (Supporting Information, Table. S2: Results of soil analysis at the beginning of the experiment). Pot distribution followed a randomised design and pot positions were changed at one time point during the experiment to take account of microclimate effects. In the case of colonisation by non-target species, pots were weeded (four times during experiment).

The response variables measured were: aboveground biomass, cover and number of individuals per plant species.

To identify treatment effects on plant community composition we assessed plant cover per species at one time point during the experiment at the time point of peak biomass development, 81 days after the first initial sowing. These estimates were performed using a modified cover estimation method following Braun Blanquet and further modified by Londo [32]. In addition to estimated cover per plant species, numbers of individuals per species were counted in each pot.

At the end of the experiment, total aboveground biomass was determined through a destructive harvest (for each of the two sowing intervals it was 78 days after the second sowing). Although the start of both sowing interval groups was at the same time, the end of the experiment was at two different time points depending on the sowing interval treatment (21-Jul-2011 & 12-Aug-2011). The different harvesting dates for these two groups (three- or six-week interval) were chosen to allow the latter sown remaining PFTs to have the same time to develop in both sowing interval treatment groups. At harvest aboveground plant parts were cut 2 mm above the soil surface, separated into PFTs, and oven-dried at 70°C to constant weight. For the first harvesting date (21-Jul-2011) only one of the three control replicates was harvested, leaving the remaining two for the second harvesting date. In addition, soil samples were taken from each pot to evaluate the nutrient status for nitrate, nitrite, ammonia, phosphorus and potassium. Measurements were performed after extraction with an 1 M KCl solution and following measurement in a Dionex ICS-3000 (except for potassium which was analysed in an 0.1 M CaCl solution with an ICP-OES). Total carbon and nitrogen in the soil were measured using an element analyser (VarioelCube, Elementar).

### Statistical Analysis

The experiment was multi-factorial in design with three main factors: priority effect of arrival order, sowing interval and density. The priority effect factor had four different levels (F-first, G-first, L-first and control sown at the same time). The sowing interval factor had two levels (three- and six weeks between early sowing of first PFT and subsequent sowing of the other two PFTs). The density factor had three levels (1.5, 2.5 and 5 g m^−2^ seeds sown). Response variables included aboveground biomass at community level and at population level plant cover per species, to assess species composition but also community structure.

Number of plant individuals per pot were analysed using a one-way ANOVA testing for the effects of density and sowing interval independently. Treatment levels were tested against each other by performing Tukey contrasts. This method enabled us not only to test for general treatment effects but to test each single level of a treatment specifically in relation to each other without increasing the chance of a type one statistical error.

Communities’ similarities were depicted by a dendrogram resulting from a hierarchical cluster analysis on the basis of a distance matrix (between group linkages). Distances were calculated on behalf of individual species’ occurrence and cover by using Pearsons’ correlation coefficient.

Biomass data was analysed using three-way ANOVA testing for effects of the factors PE, sowing interval and density as well as any interaction effects between these factors (for ANOVA Table see Table. 1). The experimental design was almost balanced and orthogonal for the three factors, except that for the three controls replicates (i.e. all PFTs sown at same time), one replicate was harvested at the first harvesting date and the remaining two at the second harvesting date. Data was generally analysed using Type III ANOVA but also using Type I ANOVA. Type I ANOVA allows to alter the order and thereby take into account the relative variability explained by this factor (see Oelmann et al. [33]) depending on when it is fitted in the model. Type I allowed us to therefore test relative effects of the three factors, depending on when they were fitted in the model.

**Table 1:** ANOVA table for the effects of experimental treatments on aboveground biomass.

Treatment	S.S.	d.f.	M.S.	F	p	Partial-η^2^
PE	1.667	3	.556	82.527	.000	.813
Sowing_Interval	.399	1	.399	59.313	.000	.510
Density	.075	2	.037	5.567	.006	.163
PE * Sowing_Interval	.151	3	.050	7.466	.000	.282
PE * Density	.040	6	.007	.983	.445	.094
Sowing_Interval * Density	.004	2	.002	.307	.737	.011
PE * Sowing_Interval * Density	.038	6	.006	.937	.476	.090
Error	.384	57	.007			

ANOVA table for effects of the experimental treatments (arrival order (PE), sowing density (density) and sowing interval) and their interactions on aboveground biomass production. Effect sizes are calculated as partial η^2^.

Normal distribution of the residuals and homogeneity of variance were checked with pp-plots and Levene’s tests respectively. Any data that did not fulfil the assumption of homogeneity of variance and normal distribution of the residuals were transformed (log 10) before analysis. Effect sizes for each factor as the proportion of explained variance were calculated as partial η^2^. Analyses were run using PASW Statistics 18 (formally known as SPSS; IBM).

## Results

### Priority Effect of PFTs on Aboveground Productivity

**Figure 2:**
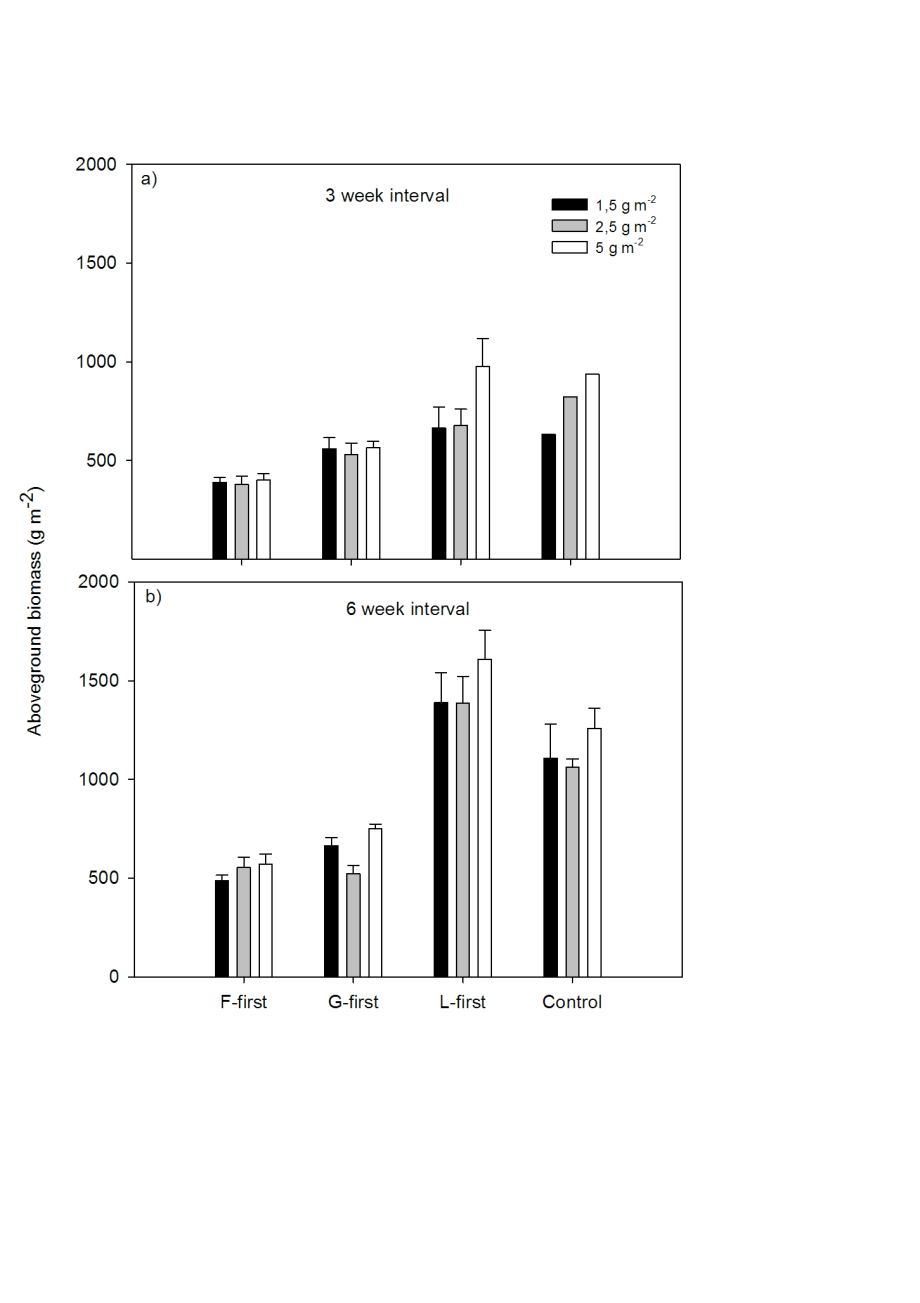
Sowing legumes first (L-first) produced the highest aboveground biomass, especially in the six-week interval treatment. Density had weaker effects on biomass than the priority effect treatment or sowing interval. Data show mean aboveground biomass (±1 SE) in the priority treatment separated into the three density levels. This is shown per sowing interval treatment with panel a) describing the three-week sowing interval and panel b) the six- week sowing interval treatment. For the priority effects treatment F, G and L-first = forbs-, grasses- and legumes-first. Replicates are n = 4 for main treatments and n = 2 or 1 for the controls in the six-week interval and three-week interval respectively.

The early sowing of one PFT (PE treatment) had a significant effect on aboveground plant productivity (Fig.2; F_ (3, 57)_ = 82.527, P<0. 0001).

Within the levels of the priority effect treatment, communities in which legumes were sown first (L-first) were the most productive (especially when sown at high density) with aboveground biomass ranging from 664±92 g m^−2^ to 1608±126 g m^−2^ followed by G-first (ranging from 521±37 g m^−2^ to 751±19 g m^−2^) and F-first (ranging from 389±20 g m^−2^ to 570±44 g m^−2^). The L-first treatment with the densities 1.5 g m^−2^, 2.5 g m^−2^ and 5 g m^−2^ being on average 25.3%, 30.5%, 27.8% more productive than their respective controls in the six week interval treatment. The treatments with a three-week sowing interval and L-first were on average 4.9% more productive in the 1.5 g m^−2^ density and 4.0% more productive within the 5 g m^−2^ density than their respective control, whereas there was no increase in productivity at 2.5 g m^−2^ (Fig. 2). Our experimental design was fully balanced except for the controls, i.e. all PFTs sown at same time, where we had three control replicates but one replicate was harvested at first harvesting date for the three-week sowing interval and the remaining two at the second harvesting date for the six-week sowing interval. This made sure that we allowed each plant community the same amount of time to develop after sowing. Taking this into account, the increase in productivity of the L-first group over that of the controls seemed not to be different between the three-week interval treatments (but no replication) but was significant in the six-week interval (F_ (3, 38)_ = 74.847, P<0. 0001).

Interactions were found between the factors priority effect and sowing interval (F_ (3, 57)_ = 7.466, P<0. 0001, see Table. 1). As a consequence, a Type I ANOVA was performed showing that irrespective of the sequence in which the other factors were fitted to the model, priority effect remained significant (F_ (3, 57)_ = 67.935, P<0. 0001).

### Species & PFT Relative Abundances

**Figure 3:**
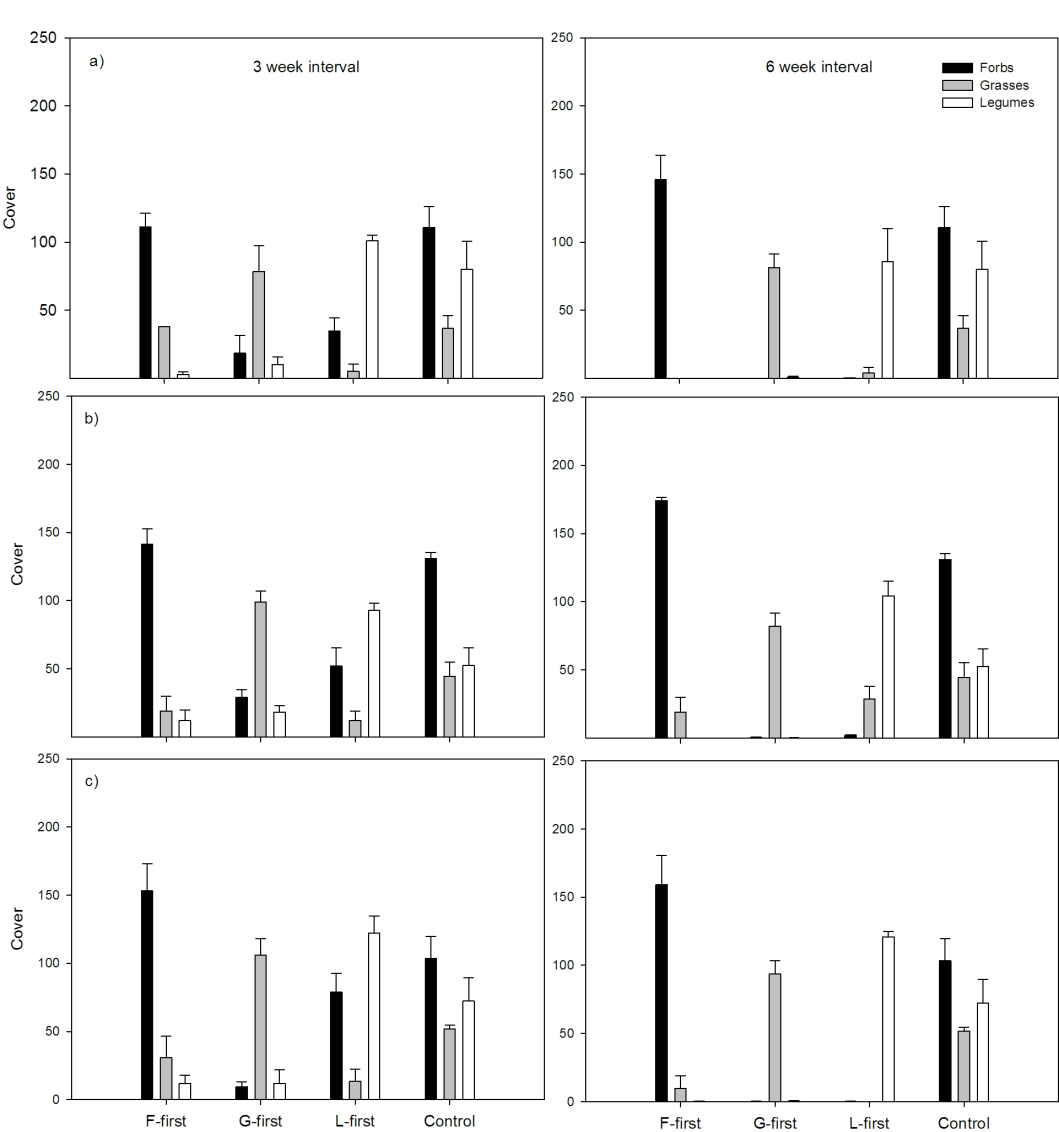
Effects of early sowing of one PFT (F, G and L-first = forbs-, grasses- and legumes-first) on the functional composition of communities in pots. Relative cover of PFTs (forbs, grasses, legumes) in pots were derived from individual species cover values summed and are depicted separately for each of the three densities: (a) 1.5 g m^−2^, (b) 2.5 g m^−2^ and (c) 5 g m^−2^ for both sowing intervals (three and six weeks, in vertical columns) from vegetation assessments at peak biomass development. The data show mean values (±1 SE); n = 4 for all treatments (except for controls where n = 3).

The PFT sown first always dominated the functional composition of the plant community. Nevertheless, there was a clear difference between treatments with a three-week interval and a six-week interval. For treatments with a six-week sowing interval the relative abundance of the PFT sown first was nearly always >90% except for one case (L-first treatment with a density of 2.5 g m^−2^ (78.6%)). The three-week interval treatment showed a more balanced relative abundance of PFTs. Priority effects on PFT abundance were consistent among the three density levels, favouring the PFT sown first with 73–84% relative abundance of forbs when forbs were sown first, 67–83% for grasses when grasses were sown first and 59–72% for legumes when legumes were sown first (Fig.3). Control treatments with simultaneous sowing showed forbs with relative abundances between 44–59%, grasses between 15–23% and legumes between 22–41%. Highest relative abundances in each PE-group were always in highest densities except for the L-first treatment where highest PFT relative abundance (72%) was at 1.5 g m^−2^.

**Figure 4:**
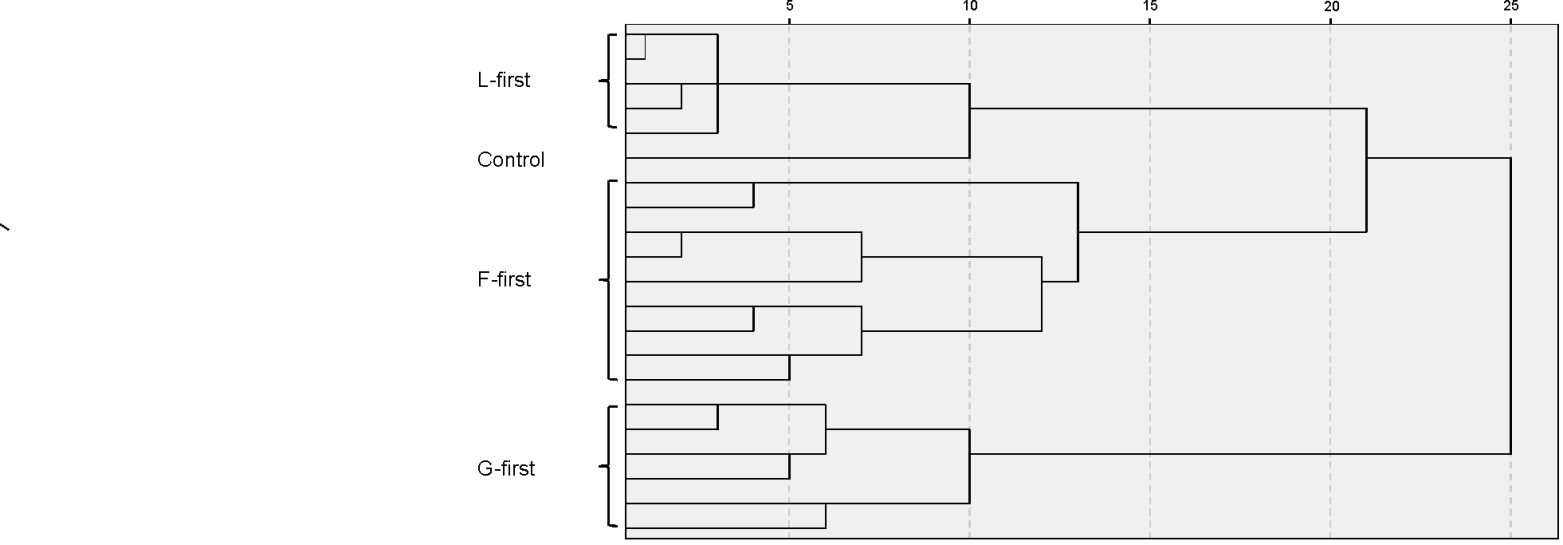
Effect of experimental treatments on the similarity of the resulting communities. Dendrogramm showing between group linkages for all replicates of the treatment groups: density (1.5, 2.5 and 5 g m^−2^; sowing interval (3- and 6- week) and the priority effects treatment F, G and L-first = forbs-, grasses- and legumes-first as a result of a cluster analysis performed on data for relative individual species cover of every single pot in June 2011. As a measure of distance between groups Pearsons correlation coefficient was taken.

Within the L-first treatment subsequently-sown PFTs (grasses and forbs) were able to establish themselves better alongside the PFT sown first (legumes) compared to the other treatments (F- or G-first) where subsequently sown PFTs were suppressed (Fig. 3). This effect was stronger in the shorter sowing interval of three-weeks. Community development was clearly affected by the priority treatment and communities having the same starting PFT were more similar than those with different starting PFTs. A cluster analysis based on data on single species cover from vegetation assessments revealed three main groups in terms of species composition, and that these groups were mainly influenced by the starting PFTs. Most differences were found between communities with G-first and the rest, followed by a separation of the F-first group and a combined L-first and control group (Fig. 4).

### Change in Soil Chemistry

Soil phosphate, nitrate and potassium were depleted by the end of the experiment when compared to values from the beginning of the experiment (T-test P<0. 05; for details see Supporting Information, Table S4: Results of the T-test as a comparison of soil nutrient levels at the beginning and at the end of the experiment). In comparison to the nutrient content of soil samples collected at time zero, C/N ratios were higher at the end of the experiment than at the beginning (t_(82)_ = 2.773, P<0.05). However, no experimental treatments had any significant effects on the measured soil variables (for details see Supporting Information, Table S3: ANOVA performed on the effect of PE-treatment on soil variables).

### Effect of Density on Aboveground Productivity

Density had a significant effect on aboveground productivity (Fig. 2; F_ (2, 57)_ = 5.567, P<0.05) with a slightly higher productivity for the higher density levels. Nevertheless within the PE- and sowing interval treatments only a few treatments showed differences in aboveground biomass as a consequence of varying density.

For the L-first treatments and the three-week sowing interval, contrasts showed that the 5 g m^−2^ treatment had a significantly higher aboveground biomass compared to the lower sowing densities (t_(9) = _2.143, P<0.05). Within the G-first treatment similar biomass yields were found in all densities in treatments with a three-week interval but not in the six-week interval. Here the 2.5 g m^−2^ group was significantly less productive (t_(9)_ = −3.975, P<0.005) than the other two density levels.

**Figure 5:**
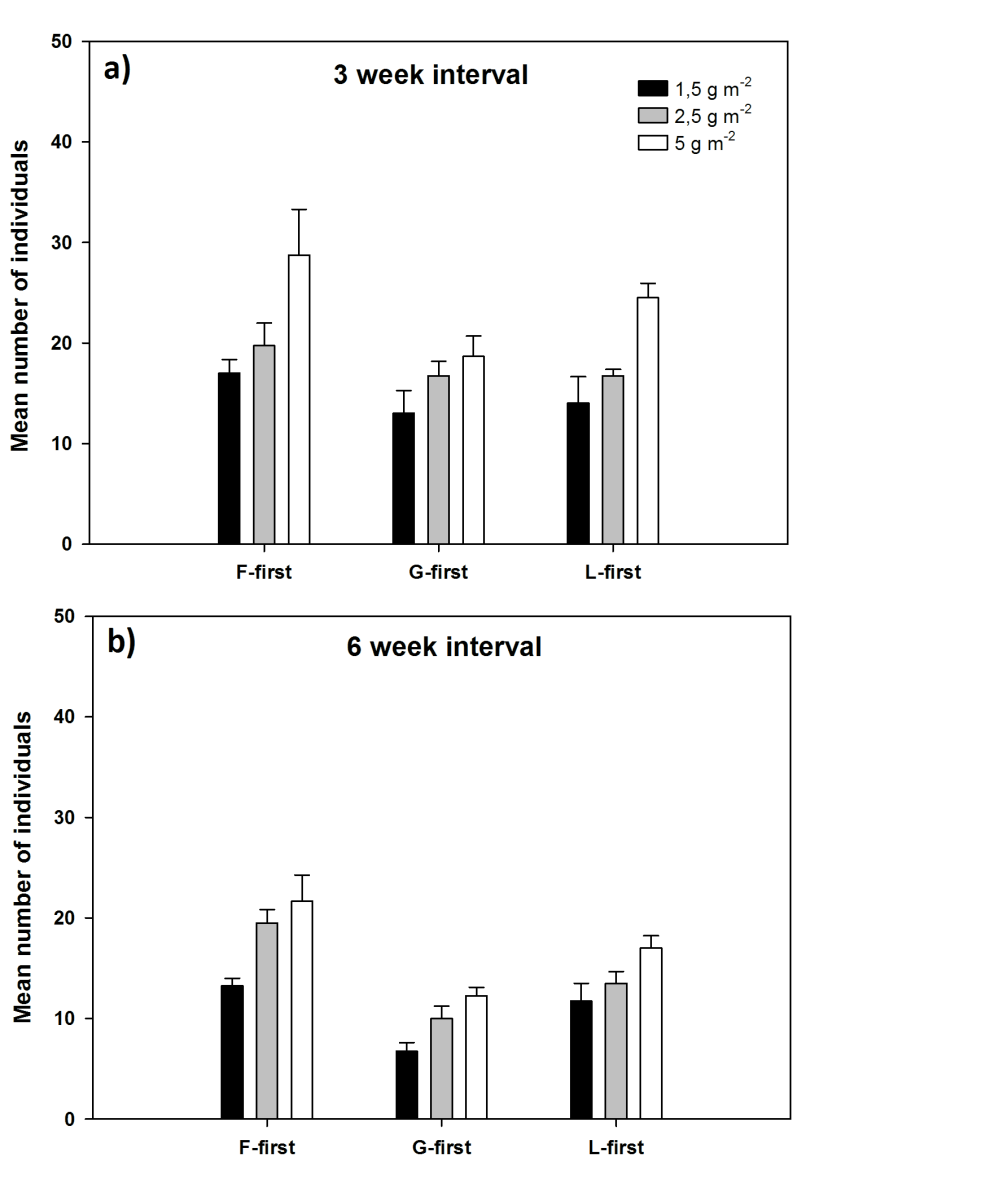
Effects of density and sowing interval on number of plant individuals per pot. For the priority effects treatment (PE) F, G and L-first = forbs-, grasses- and legumes-first. Values are mean number of plant individuals per pot with the PFT sown first on the x-axis and for all three densities for both groups sown with a three week interval (a) and a six week interval (b) between sowing of the first and remaining PFTs. (n = 4; ±1 SE).

The density treatment also influenced the number of individuals per pot, insofar as with increasing density the mean number of individuals increased across all treatments. Treatments with a sowing density of 1.5 g m^−2^ were having the lowest mean number of individuals (t_(66)_ = 4.200, P<0.001) and treatments with a sowing density of 5 g m^−2^ were having significantly higher numbers of individuals (t_(66)_ = 2.841, P<0.005) for both sowing interval treatments (Fig.5).

The influence of the priority effect-treatment was also visible in terms of numbers of individuals and showed the same trend for both sowing intervals. Treatments with a six-week interval had fewer individuals in each density level than in the three-week interval treatment (t_ (67)_ = 3.846, P<0.001; Fig. 5).

### Sowing Interval Effects

The effect of sowing interval on aboveground productivity between sowing of the first- and subsequent PFTs was significant (Fig. 2; F_ (0.399)_ = 59.313, P<0. 0001), with a sowing interval of six weeks showing increased productivity across all levels of the density treatment compared to the three-week interval. On average all treatments within the six-week interval group were more productive than the groups with a three-week sowing interval. The most pronounced difference in aboveground biomass was visible for the L-first treatment. In comparison (all sowing densities together) the L-first treatment with a six-week interval had 89% more biomass compared to the three-week interval group. The other PE groups for F- and G-first showed 38% and 16% increase in aboveground biomass respectively in comparison to the treatments with a three-week interval. Simultaneously sown controls were on average 62% more productive in the six-week interval group compared to the three-week interval control treatments.

The sowing interval also had strong effects on overall number of individuals per pot (t_(76)_ = 3.588, P>0.005; Fig. 5) and the overall plant species richness (t_(76)_ = 4.376, P>0.001) with lower individual and species numbers in the six week sowing interval.

## Discussion

Our study found that priority effects of order of arrival at plant functional level had a substantial effect on aboveground productivity of sown communities, with L-first treatment being more productive than controls sown at the same time or grass or forb species sown first (Figures. 2). These results (see Figure 4) confirm findings of Körner et al. (2008) and Ejrneas et al. (2006) in that the order of arrival of functionally different groups was critical to the development of their experimental communities resulting in clusters of different floristic distances to one another. In our study this is clearly reflected by the cluster analysis (Figure 4) on the basis of species’ occurrence and relative abundances and the correlation to the functional composition of the resulting community. This analysis shows that the strong separation of communities was dependant on the starting PFTs which underlines the importance of priority effects in influencing the assembly process as found in a number of other studies [10], [11], [13]. Körner et al. [10] found that in terms of biomass production and final functional group composition after two growing seasons the L-first treatments and simultaneously sown controls were the most similar. Our experiment confirms this, even if our study ran for a shorter length of time and with a different species pool.

Our cover data confirm the aboveground biomass data in that in the L-first treatment, the functional groups were present in more balanced abundances than when grasses or forbs were sown first. Nevertheless, in the L-first treatment legumes contributed more to the overall community biomass than the other starting PFTs when they were sown first. L-first treatments were more productive than the other priority treatments irrespective of the sowing interval or sowing density, despite the fact that forbs were very dominant in relative cover and mean number of individuals. This suggests that legumes were better able to get a head-start and be productive before the later groups arrived even though species richness of the communities was rather modulated by the sowing interval (lower species richness when sowing interval was bigger) than by the identity of the species sown first, their traits allowed for better subsequent establishment of non-legume PFTs. In our experience legume species often do compete well and grow quickly in initial stages of experiments, as well as allowing for N facilitation with neighbours. Although legumes may not arrive earlier than other functional groups in naturally assembling communities, in ecological restoration we often wish to direct succession onto a desirable trajectory [34].

It seems that sowing legumes first led to asymmetric competition and fast growth of legumes [35] but at the same time more functional complementarity occurred between legumes and the other PFTs. A possible mechanism is the smaller rooting system (root mass fraction) of legumes if they are actively fixing atmospheric nitrogen, such that subsequent PFTs have more opportunities for both root space and nutrient foraging (also known as N sparing, [36]) and hence overall productivity is stimulated. Over a longer time span and under field conditions however, N facilitation (whereby the neighbours of N_2_-fixers profit from legume-fixed N) may also cause higher productivity [37]. In this experiment treatments did not affect soil chemistry significantly even if C/N ratios changed from the beginning to the end of the experiment.

A likely explanation for the strong presence of forbs (at least considering cover and species numbers; Figures. 3&5) could be that forbs were overrepresented in species number right from the beginning (compared to the other PFTs there were 14 species sown within this functional group and only 7 for each legumes and grasses) to reflect the species and PFT composition common for restored grasslands in central Europe. But thus the F-first treatment had the lowest aboveground productivity, at least for our study no positive relationship between cover and productivity could be confirmed in this case (compare [27]).

We could not confirm the hypothesis that the longer the sowing interval the lower the aboveground productivity of the system will be. We hypothesised also that this would be because of stronger asymmetric competition between PFTs when early arrivers get a head-start and very little complementarity between PFTs can occur. What we found instead was that communities with a six-week sowing interval were more productive than those with a three-week interval (Figure. 2) despite the data showing higher mean species numbers (and also a higher species richness) in pots with a three-week sowing interval. A likely explanation would be that the starting PFT in the six-week interval group had three weeks longer to establish itself and grow than the three-week interval group. While the timespan for the two interval groups was the same after the second sowing occurred, meaning that for the two subsequently sown PFTs in every treatment the time allowed for growing was similar, the PFT sown first had 3 weeks more time to develop within the six-week interval. In general, later arriving PFTs contributed less towards community biomass as a consequence of the PE treatment and this makes sense since competitive advantage of the PFT sown first and thus asymmetric competition is part of the expected priority effect. Kardol et al. (2013) postulated that a priority-driven competitive advantage of early arriving species over later arriving species affected the probability of species coexistence and led to reduced species richness through competitive exclusion. This corresponds to our findings as we could also show a reduced number of individuals and lower plant species richness in the six week interval groups compared to the three week interval groups indicating the suppression of later arriving species by the PFTs sown first.

This could also be seen by looking at relative PFT contributions for the three- and the six-week interval (Fig. 3) where the six-week interval treatment was always particularly dominant without substantial contribution by the later sown functional groups species. We consider the starting PFT had a competitive advantage of arriving first and having better access to resources (especially light) before the competition with later arriving species occurred. As a result, niche space was likely filled more efficiently by the PFT sown first in the longer sowing interval treatments resulting in lower resource availability for later arriving plants as observed in other systems [38]. For a sowing interval of six weeks we observed an intensified dominance of the PFT sown first which was almost always above 90% in relative abundance at peak biomass whereas in the three-week interval, later sown PFTs were still able to compete and sustain a higher proportion within the communities.

Our hypothesis stated that because of the law of constant yield, sowing different seed densities will result in higher individual numbers at higher sowing densities, but overall aboveground productivity will remain the same across the different densities. Aboveground productivity did not differ across the density treatment but at the same time mean number of individuals per pot were significantly higher in treatments with higher sowing densities (Fig. 5). However, this difference did not strongly affect aboveground biomass and this suggests that soil nutrients were fully exploited by the community independent of how many individuals were present. As a consequence, higher sowing densities did not result in higher overall aboveground biomass, possibly because each individual was not able to be as productive as in lower density treatments, which is consistent with the law of constant yield [25]. It seems that the duration of our experiment was long enough for the law of constant yield to take effect.

## Conclusion

The influence of assembly history on aboveground productivity was much stronger than sowing density or sowing interval (see Table 1 showing different effect sizes of factors). PE treatments led to the development of differently structured plant communities in terms of plant functional composition and dominance structure (Figs. 3&4). In natural succession plants often follow a sequence in which certain species establish and represent the community at a certain time point. This is often controlled by the local species pool and the availability of suitable environmental conditions for establishment. In our experiment both determinants were excluded (as often done in restoration practices) in the setup and thus the observed priority effect is of purely artificial nature. An important aspect of the priority effect was that the PFT sown first had significant effects on further functional composition with strong dominance of the early arriving PFT in the community. Although a larger sowing interval led to higher asymmetric competition we found evidence for complementarity between PFTs in the three-week interval treatment. In the latter, the cover of later arriving PFTs was larger than for other treatments when legumes were sown first, suggesting that the optimal combination of functional groups would be sowing legumes first but making sure the sowing interval was not too long to enable the plants to achieve full maturity and thus have negative impacts on newcomers. Our results indicate that priority effects affect community development and function and that the severity of this impact seems to be much more driven by the question “Who comes first (and what is their function)?” than by questions like “when?” or “how many?”. A possible application for our results can be found within the fields of restoration or agricultural practise when it comes to restoring ecosystem services or to increase productivity in low input high diversity systems [39], [40]. To what extend we are able to set direction and to influence the development of plant communities via priority effects and their potential to create alternative stable states within plant communities is still to be addressed. So far to our knowledge no field experiments have tested these priority effects of functional group arrival time on community assembly and this would include a longer term and of course larger-scale assessment of priority effects on structure and function of communities. We are currently addressing this in a field experiment with the same kind of PFT-first treatments as in this pot experiment, where that we also find priority effects of sowing legumes early, even if one allows other species to invade aside from the sown species. Our study nevertheless confirms previous concepts of legumes as keystone species within N-limited grassland habitats, since the legumes seemed to have the ability to dominate at the same time as interacting with other groups in a complementary way [29], [41]. Other studies have proposed asymmetric competition and plant-soil feedback effects as possible mechanisms behind priority effects (e.g. Grman and Suding 2010). Our study emphasises the need to also consider N facilitation effects of legumes as a driver of priority effects.

## Supporting Information

Table S1 **Plant species per functional group with respective seed mass per pot.**
(DOCX)

Table S2 **Results of soil analysis at the beginning of the experiment.**
(DOCX)

Table S3 **ANOVA performed on the effect of PE-treatment on soil variables.**
(DOCX)

Table S4 **Results of the T-test as a comparison of soil nutrient levels at the beginning and at the end of the experiment.**
(DOCX)
