# Supplementary material for: Priority Effects of Time of Arrival of Plant Functional Groups Override Sowing Interval or Density Effects: A Grassland Experiment
Source: PLoS One. 2014 Jan 31;9(1):e86906. doi: 10.1371/journal.pone.0086906 (PMC3908951; doi:10.1371/journal.pone.0086906)
Supplement: Table S1 — Plant species per functional group with respective seed mass per pot. (DOCX) [file pone.0086906.s001.docx]

**Supporting Information**

Supplementary Table **S1**: Plant species per functional group with respective seed mass per pot.

| **Plant functional group** | **Plant Species** | **seed mass in pots with 1,5 g/m^2^(g)** | **seed mass in pots with 2,5 g/m^2^(g)** | **seed mass in pots with 5 g/m^2^(g)** |
| --- | --- | --- | --- | --- |
| Forb | Achillea millefolium | 0,0007 | 0,0012 | 0,0023 |
| Forb | Centaurea jacea jacea | 0,0007 | 0,0012 | 0,0025 |
| Forb | Crepis biennis | 0,0003 | 0,0005 | 0,0009 |
| Forb | Agrimonia eupatoria | 0,0160 | 0,0266 | 0,0532 |
| Forb | Betonica officinalis | 0,0023 | 0,0038 | 0,0076 |
| Forb | Dianthus carthusianorum | 0,0018 | 0,0029 | 0,0058 |
| Forb | Knautia arvensis | 0,0014 | 0,0023 | 0,0047 |
| Forb | Leontodon autumnalis | 0,0007 | 0,0012 | 0,0025 |
| Forb | Leucanthemum vulgare | 0,0007 | 0,0012 | 0,0023 |
| Forb | Falcaria vulgaris | 0,0003 | 0,0005 | 0,0011 |
| Forb | Plantago lanceolata | 0,0011 | 0,0019 | 0,0037 |
| Forb | Origanum vulgare | 0,0004 | 0,0006 | 0,0012 |
| Forb | Stachys recta | 0,0016 | 0,0026 | 0,0053 |
| Forb | Galium verum | 0,0009 | 0,0015 | 0,0029 |
| Legume | Medicago falcata | 0,0014 | 0,0023 | 0,0047 |
| Legume | Lotus corniculatus | 0,0013 | 0,0021 | 0,0042 |
| Legume | Medicago lupulina | 0,0023 | 0,0039 | 0,0077 |
| Legume | Securigera varia | 0,0002 | 0,0004 | 0,0008 |
| Legume | Trifolium campestre | 0,0005 | 0,0009 | 0,0018 |
| Legume | Trifolium pratense | 0,0006 | 0,0011 | 0,0021 |
| Legume | Trifolium arvense | 0,0014 | 0,0023 | 0,0047 |
| Gras | Anthoxanthum odoratum | 0,0021 | 0,0035 | 0,0070 |
| Gras | Arrhenatherum elatius | 0,0028 | 0,0047 | 0,0095 |
| Gras | Briza media | 0,0098 | 0,0163 | 0,0327 |
| Gras | Dactylis glomerata | 0,0007 | 0,0012 | 0,0023 |
| Gras | Bromus erectus | 0,0020 | 0,0033 | 0,0065 |
| Gras | Helictotrichon pratense | 0,0053 | 0,0088 | 0,0175 |
| Gras | Poa angustifolia | 0,0008 | 0,0013 | 0,0026 |
