# Supplementary material for: Priority Effects of Time of Arrival of Plant Functional Groups Override Sowing Interval or Density Effects: A Grassland Experiment
Source: PLoS One. 2014 Jan 31;9(1):e86906. doi: 10.1371/journal.pone.0086906 (PMC3908951; doi:10.1371/journal.pone.0086906)
Supplement: Table S2 — Results of soil analysis at the beginning of the experiment. (DOCX) [file pone.0086906.s002.docx]

**Supporting Information**

Supplementary Table S2: Results of soil analysis at the beginning of the experiment.

| Total Carbon (%) | Total Nitrogen (%) | Ammonia (%) | Nitrite (%) | Nitrate (%) | Phosphate (%) | Potassium (μg/kg) |
| --- | --- | --- | --- | --- | --- | --- |
| 3,273 | 0,076 | < 0,0005 | < 0,0005 | 0,036 | 0,005 | 90,000 |
