# Supplementary material for: Priority Effects of Time of Arrival of Plant Functional Groups Override Sowing Interval or Density Effects: A Grassland Experiment
Source: PLoS One. 2014 Jan 31;9(1):e86906. doi: 10.1371/journal.pone.0086906 (PMC3908951; doi:10.1371/journal.pone.0086906)
Supplement: Table S3 — ANOVA performed on the effect of PE-treatment on soil variables. (DOCX) [file pone.0086906.s003.docx]

**Supporting Information**

Supplementary Table S3: ANOVA performed on the effect of PE-treatment on soil variables.

|  | SS | | df | MS | F | Sig. |
| --- | --- | --- | --- | --- | --- | --- |
| Nitrite (%) | Between Group | ,000 | 3 | ,000 | ,785 | ,506 |
|  | Within Group | ,000 | 77 | ,000 |  |  |
|  | Total | ,000 | 80 |  |  |  |
| Nitrate (%) | Between Group | ,000 | 3 | ,000 | 1,811 | ,152 |
|  | Within Group | ,000 | 77 | ,000 |  |  |
|  | Total | ,000 | 80 |  |  |  |
| Ammonium (%) | Between Group | ,000 | 3 | ,000 | . | . |
|  | Within Group | ,000 | 77 | ,000 |  |  |
|  | Total | ,000 | 80 |  |  |  |
| Potassium (%) | Between Group | 8,296 | 3 | 2,765 | ,918 | ,436 |
|  | Within Group | 231,903 | 77 | 3,012 |  |  |
|  | Total | 240,199 | 80 |  |  |  |
| Phosphate (µg/g) | Between Group | ,000 | 3 | ,000 | . | . |
|  | Within Group | ,000 | 77 | ,000 |  |  |
|  | Total | ,000 | 80 |  |  |  |
| Total C (%) | Between Group | 1,936 | 3 | ,645 | ,672 | ,572 |
|  | Within Group | 73,944 | 77 | ,960 |  |  |
|  | Total | 75,880 | 80 |  |  |  |
| Total N (%) | Between Group | ,000 | 3 | ,000 | ,191 | ,902 |
|  | Within Group | ,030 | 77 | ,000 |  |  |
|  | Total | ,030 | 80 |  |  |  |
| C/N | Between Group | 125,782 | 3 | 41,927 | 2,316 | ,082 |
|  | Within Group | 1393,798 | 77 | 18,101 |  |  |
|  | Total | 1519,580 | 80 |  |  |  |
