# Supplementary material for: Priority Effects of Time of Arrival of Plant Functional Groups Override Sowing Interval or Density Effects: A Grassland Experiment
Source: PLoS One. 2014 Jan 31;9(1):e86906. doi: 10.1371/journal.pone.0086906 (PMC3908951; doi:10.1371/journal.pone.0086906)
Supplement: Table S4 — Results of the T-test as a comparison of soil nutrient levels at the beginning and at the end of the experiment. (DOCX) [file pone.0086906.s004.docx]

**Supporting Information**

Supplementary Table S4: Results of the T-test as a comparison of soil nutrient levels at the beginning and at the end of the experiment.

|  | **Test for independent variables** | | | | | | | |
| --- | --- | --- | --- | --- | --- | --- | --- | --- |
|  | | Levene-Test | | T-Test | | | |  |
|  |  | F | Sig. | t | df | Sig. (2-sided) | |  |
| Nitrite (%) | Homogeneity assumed | ,150 | ,699 | -,191 | 82 | ,849 |  |  |
|  | Homogeneity not assumed |  |  | -1,000 | 80,000 | ,320 |  |  |
| Nitrate (%) | Homogeneity assumed | 501,393 | ,000 | 48,994 | 82 | ,000 |  |  |
|  | Homogeneity not assumed |  |  | 7,842 | 2,000 | ,016 |  |  |
| Potassium (µg/g) | Homogeneity assumed | 95,307 | ,000 | 40,649 | 82 | ,000 |  |  |
|  | Homogeneity not assumed |  |  | 7,371 | 2,001 | ,018 |  |  |
| Phosphate (µg/g) | Homogeneity assumed | 444,885 | ,000 | 105,239 | 82 | ,000 |  |  |
|  | Homogeneity not assumed |  |  | 16,737 | 2,000 | ,004 |  |  |
| Total C (%) | Homogeneity assumed | ,415 | ,521 | ,301 | 82 | ,765 |  |  |
|  | Homogeneity not assumed |  |  | ,376 | 2,247 | ,739 |  |  |
| Total N (%) | Homogeneity assumed | ,419 | ,519 | -,799 | 82 | ,426 |  |  |
|  | Homogeneity not assumed |  |  | -,902 | 2,196 | ,455 |  |  |
| C/N | Homogeneity assumed | 3,890 | ,052 | 2,773 | 82 | ,007 |  |  |
|  | Homogeneity not assumed |  |  | 10,987 | 10,566 | ,000 |  |  |
